# Fever of Unknown Origin: A Rare Presentation of Parathyroid Carcinoma

**DOI:** 10.7759/cureus.75868

**Published:** 2024-12-17

**Authors:** Keshao B Nagpure, Sunita Kumbhalkar, Amol Dube, Sake Indu

**Affiliations:** 1 General Medicine, All India Institute of Medical Sciences, Nagpur, Nagpur, IND

**Keywords:** fever of unknown origin, hypercalcemia, parathyroid adenoma, parathyroid carcinoma, primary hyperparathyroidism

## Abstract

Fever of unknown origin (FUO) can be a common manifestation of multiple disease processes like infections, hematological & solid organ malignancies, autoimmune disorders, and autoinflammatory diseases. Endocrine causes of FUO are rare but should be considered in differential diagnosis. We present a case of a 35-year-old female with prolonged on-and-off fever and intermittent vomiting for nine months, where extensive workups for chronic infections, malignancy, and autoimmune conditions initially yielded no definitive diagnosis. Upon further investigation at our institution, the patient was found to have elevated serum calcium levels, nephrocalcinosis on ultrasound, and an extremely high parathyroid hormone level (2810 pg/ml), which directed attention toward a parathyroid disorder. A neck ultrasound revealed a right inferior parathyroid adenoma, which was confirmed by single-photon emission computed tomography (SPECT) imaging. The patient underwent parathyroidectomy, followed by postoperative hypocalcemia, which was promptly managed. Histopathological examination confirmed parathyroid carcinoma. The patient was subsequently referred for oncological care and is under follow-up. This case highlights the importance of considering endocrine causes, such as parathyroid carcinoma, in the workup of FUO, even in the absence of typical symptoms.

## Introduction

Fever of unknown origin (FUO) is a common clinical and diagnostic challenge, typically caused by infections or malignancies. Thyroid and parathyroid disease is rarely reported as a cause of FUO. Primary hyperparathyroidism (PHPT) is a condition characterized by hypercalcemia resulting from the excessive, uncontrolled secretion of parathyroid hormone (PTH). It is most commonly caused by benign parathyroid adenomas, although parathyroid carcinoma, a rare and often aggressive malignancy, can also be implicated. PHPT typically presents with severe hypercalcemia, along with skeletal and renal complications such as subperiosteal cysts, brown tumors, a "salt and pepper" appearance of the skull, and nephrocalcinosis. Neuropsychiatric symptoms, including gait disturbances, muscle atrophy, and psychosis, may also occur but are less common [1]. In some cases, atypical presentations, such as fever of unknown origin (FUO), can delay diagnosis and complicate patient management. PHPT remains underdiagnosed in developing countries due to limited access to routine serum biochemical screening, leading to later-stage diagnoses and a higher incidence of classic complications such as bone disease and renal dysfunction. In these regions, PHPT often affects women at a younger age. The disease-specific mortality rate for parathyroid carcinoma is 7.4%, with recurrence and persistence rates of 4.16% and 2.17%, respectively [2]. This report highlights a case of parathyroid carcinoma in a young woman with hypercalcemia and FUO, underscoring the importance of including parathyroid adenoma and carcinoma in the differential diagnosis of FUO.

## Case presentation

A 35-year-old female was referred to our tertiary care hospital for evaluation of fever of unknown origin (FUO). She had experienced intermittent fevers for nine months, accompanied by sporadic vomiting. On presentation, she was afebrile, with a pulse of 78/min and blood pressure of 130/80 mmHg. Physical examination was unremarkable. She had intermittent fever spikes, with recorded temperatures ranging from 101.4°F to 102.2°F.

Over the period of nine months, she had sought multiple consultations. Her previous diagnostic evaluation is as given below.

Complete blood count, blood sugars, serum electrolytes, liver and kidney function tests, and thyroid stimulating hormone (TSH) were within normal limits. However, serum alkaline phosphatase was markedly raised (2693 U/L; normal range: 40-129 U/L ). The erythrocyte sedimentation rate was raised (34 mm after the first hour); however, C-reactive protein was normal (1 mg/L). The urine routine and microscopy were within normal limits. Blood culture and urine culture did not show any growth of organisms. Comprehensive investigations for chronic infections, including tuberculosis, brucellosis, chronic malaria, human immunodeficiency virus (HIV) antibodies, hepatitis B surface antigen (HBsAg) and hepatitis C virus (HCV) antibodies were negative. Antinuclear antibodies and antinuclear antibody (ANA) immunoblot were negative for autoantibodies. X-ray chest did not reveal any abnormality. Ultrasounds of the abdomen and pelvis were repeated four times over a nine-month duration. The last ultrasound, one week prior, reported normal-sized kidneys with altered renal cortical echotexture/raised bands of cortical echotexture along with multiple hyperechoic foci in the poles/corticomedullary junctions, representing nephrocalcinosis. Upper gastrointestinal endoscopy showed antral gastritis. Two-dimensional echocardiography showed normal left ventricular function and there was no evidence of vegetation, clots, or pericardial effusion. MRI brain did not reveal any evidence of chronic meningitis. Bone marrow aspiration and biopsy examination revealed normal cellularity. PET-CT scan showed low-grade metabolism involving bilateral kidneys and diffuse low-grade metabolism involving the marrow of the axial and appendicular skeleton. Despite a nine-month journey of extensive consultations and exhaustive investigations, diagnosis in this patient who presented with pyrexia of unknown origin and vomiting remained elusive. Hence, the patient was referred to our tertiary care center.

As a case of classical FUO, we started reevaluating the patient, which began with a thorough clinical examination that did not reveal any abnormality except for a small palpable nodule about 1 x 1 cm in size, firm in consistency, situated in the suprasternal notch on the right side. The nodule did not move with deglutition or protrusion of the tongue. A neck ultrasound revealed a well-defined, encapsulated, heteroechoic, predominantly solid lesion measuring 1.2 x 2 cm. The lesion showed punctate calcifications and peripheral vascularity, features suggestive of a benign etiology. A thyroid scan done with 99m TC- pertechnetate showed homogenous tracer uptake in both lobes of the thyroid gland, which appeared normal in size. Scintigraphic and single photon emission computed tomography (SPECT-CT) findings were reported as parathyroid adenoma just inferior to the lower pole of the right thyroid lobe, possibly involving the right inferior group of parathyroid glands or ectopic adenoma. Further workup revealed severe hypercalcemia (14.5 mg /dl) and markedly raised parathyroid hormone (PTH) levels of 2810 pg/ml (Table 1).

**Table 1:** Preoperative, postoperative, and follow-up serum calcium and parathyroid hormone levels

Duration	Preoperative	Postoperative					Follow-up				Normal range
		Day 0	Day 1	Day 2	Day 3	Day 7	Month 1	Month 6	Year 1	Year 2	
Serum calcium	14.5	10	6.8	6.3	6.7	8.8	9.13	9.31	9.24	9.45	8.8-10.6 mg/dl
Serum parathyroid level	2810			96.34	96.34	75.87	60.2	16.68	78.2	50.1	10-65 pg/ml

**Figure 1:**
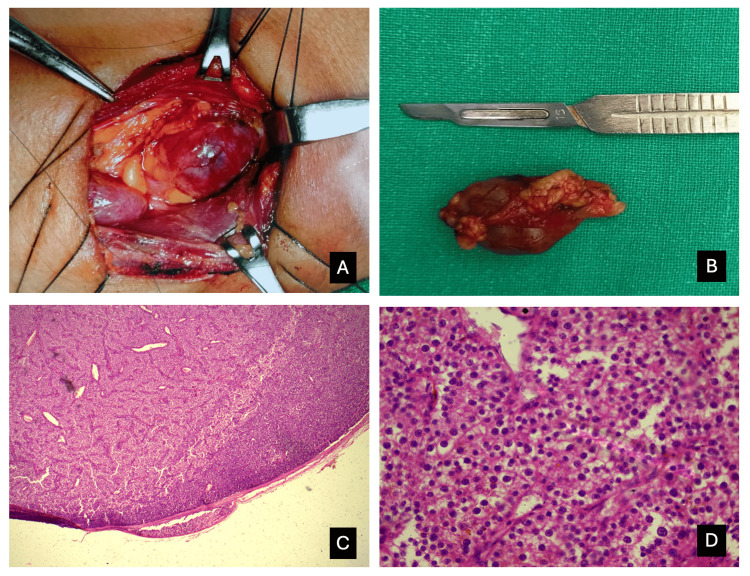
A. Intraoperative tumor mass; B. Postoperative specimen of the parathyroid tumor mass; C. Capsular invasion by tumor; D. Tumor cell morphology, abundant pale to eosinophilic cytoplasm with salt and pepper appearance of nuclei

Based on clinical, radiological, and biochemical investigations, a diagnosis of primary hyperparathyroidism caused by a parathyroid adenoma was confirmed. Severe hypercalcemia was initially treated with intravenous hydration (normal saline) and Intravenous (IV) zoledronic acid. The patient subsequently underwent parathyroidectomy. She developed postoperative hypocalcemia which was successfully managed with IV calcium. Following surgery, her fever and vomiting resolved and PTH levels gradually returned to normal within one month. However, histopathology showed capsular invasion with abundant pale to eosinophilic cytoplasm with salt and pepper appearance of nuclei suggestive of parathyroid carcinoma (Figure 1). Somatic mutation studies (CDC73) could not be done due to financial constraints. She was referred to an oncology center for further management. She has been under regular follow-up for the past two years, remaining asymptomatic with normal serum calcium (9.45 mg/dl) and serum PTH levels (50.1 pg/ml) (Table 1).

## Discussion

In 1961, Petersdorf and Beeson defined fever of unknown origin (FUO) as a persistent fever of 38.3 °C (101 °F) or higher, lasting for at least three weeks, with no identifiable cause despite one week of inpatient evaluation. With advancements in healthcare, Durack and Street later revised these criteria, reducing the investigation period to either three days of inpatient evaluation or a minimum of three outpatient visits. FUO remains a diagnostic challenge in clinical practice, with common aetiologies including infections (e.g., extrapulmonary tuberculosis), malignancies (e.g., occult cancer), autoimmune disorders (e.g., systemic lupus erythematosus), and autoinflammatory diseases (e.g., adult-onset Still's disease). A study on the epidemiology of classical fever of unknown origin (FUO) across 21 countries with varying levels of economic development revealed that infections and malignancies are the most common causes, followed by collagen vascular diseases and other miscellaneous conditions. Endocrine causes of FUO are relatively uncommon, with thyroiditis being the most frequently identified endocrine etiology; however, parathyroid disorders are not reported [3]. Primary hyperparathyroidism (PHPT) is commonly caused by parathyroid adenoma, often marked by high levels of serum calcium and PTH, nephrocalcinosis, and other systemic manifestations such as bone pain and renal dysfunction. However, parathyroid carcinoma is a rare cause of PHPT.

Our case adds to the growing body of literature on the potential for parathyroid carcinoma to present as a rare but important cause of FUO. While parathyroid adenoma has been reported in a few case studies as a rare cause of FUO, parathyroid carcinoma has not previously been identified as a potential etiology.

Clinical features of parathyroid carcinomas are like parathyroid adenoma; however, patients with parathyroid carcinomas are more likely to have symptoms, a neck mass, bone and kidney disease, marked hypercalcemia, and very high serum parathyroid hormone concentrations. As demonstrated in our patient, the presence of a palpable neck mass, nephrocalcinosis, and markedly elevated parathyroid hormone levels, far exceeding the upper limits of normal, suggested a diagnosis of parathyroid carcinoma rather than the more common benign parathyroid adenoma. However, despite these clinical clues, FUO remains a rare and often challenging presentation of parathyroid carcinoma.

As previously noted, fever in such cases is typically the result of an inflammatory response mediated by endogenous pyrogens, which act on the hypothalamus and reset the body’s thermoregulatory set point. The exact mechanism by which parathyroid carcinoma may contribute to fever remains somewhat speculative, but it is likely related to the secretion of cytokines or other inflammatory mediators by the tumor itself. Tumors such as parathyroid carcinoma, as well as other malignancies, can produce a range of endogenous pyrogens (e.g., interleukins, tumor necrosis factor-alpha) that influence the hypothalamic regulation of body temperature [4]. The observed fever in our patient could thus be attributed to this inflammatory cascade, further supporting the notion that parathyroid carcinoma should be considered as a potential cause of FUO.

In contrast to parathyroid adenoma, parathyroid carcinoma has a more unpredictable and often indolent clinical course. While most parathyroid carcinomas are diagnosed in the presence of severe symptoms, such as hypercalcemia and neck masses, some cases may remain asymptomatic or present with minimal signs until they become advanced. The slow progression of parathyroid carcinoma can often complicate its diagnosis, especially in cases where systemic manifestations are subtle, and a definitive diagnosis is delayed [5]. Our patient’s relatively indolent course, despite the severity of biochemical abnormalities, underscores this challenge.

A key aspect of managing patients with parathyroid carcinoma is early recognition and timely intervention. Given the aggressive nature of parathyroid carcinoma and its potential for local recurrence and metastasis, early surgical intervention, typically in the form of en-bloc resection, is critical. Unfortunately, the rarity of this malignancy means that diagnosis is often delayed, leading to a poorer prognosis. Our patient’s management strategy involved surgical excision of the parathyroid mass, with subsequent monitoring of calcium and parathyroid hormone levels, which showed a favorable initial response. However, long-term follow-up is essential, as recurrent or metastatic disease can occur even after successful resection. Recurrence rates can be as high as 50%, with a median disease-free interval of 36 months following the initial surgery; however, recurrences have been reported even after 15 to 20 years. Because of the potential for late recurrence, lifelong follow-up is recommended. Hypercalcemia is usually the first indication of recurrence [6].

This case emphasizes the need for clinicians to consider rare but potentially serious causes of FUO, especially in patients with suggestive clinical findings such as hypercalcemia, neck masses, and severe parathyroid hormone elevations. It also highlights the importance of recognizing the broader spectrum of parathyroid disorders and their potential to present in unusual ways. Further research into the pathophysiology of fever in parathyroid carcinoma could help elucidate the underlying mechanisms and potentially guide therapeutic approaches.

## Conclusions

Fever of unknown origin remains a challenging diagnostic entity, and parathyroid carcinoma, though rare, should be considered a differential diagnosis in patients presenting with FUO, particularly when associated with significant hypercalcemia, elevated parathyroid hormone levels, and neck masses. This case highlights the need for heightened awareness of parathyroid carcinoma as a potential cause of FUO.
